# Gut microbiome diversity within *Clostridia* is negatively associated with human obesity

**DOI:** 10.1128/msystems.00627-24

**Published:** 2024-07-16

**Authors:** Laura Salazar-Jaramillo, Jacobo de la Cuesta-Zuluaga, Luis A. Chica, María Cadavid, Ruth E. Ley, Alejandro Reyes, Juan S. Escobar

**Affiliations:** 1 Vidarium–Nutrition, Health and Wellness Research Center, Grupo Empresarial Nutresa, Medellin, Colombia; 2 Department of Microbiome Science, Max Planck Institute for Biology, Tübingen, Germany; 3 Department of Biological Sciences, Max Planck Tandem Group in Computational Biology, Research Group in Computational Biology and Microbial Ecology (BCEM), Universidad de los Andes, Bogota, Colombia; 4 Department of Pathology and Immunology, Edison Family Center for Genome Sciences and Systems Biology, Washington University School of Medicine, St. Louis, Missouri, USA; Qingdao Institute of Bioenergy and Bioprocess Technology, Chinese Academy of Sciences, Qingdao, Shandong, China

**Keywords:** 16S rRNA, dnaK, gyrB, BMI, metagenomics, *Lachnospiraceae*, *Ruminococcaceae*, *Oscillospiraceae*, *Acutalibacteraceae*, *Vescimonas*

## Abstract

*Clostridia* are abundant in the human gut and comprise families associated with host health such as *Oscillospiraceae,* which has been correlated with leanness. However, culturing bacteria within this family is challenging, leading to their detection primarily through 16S rRNA amplicon sequencing, which has a limited ability to unravel diversity at low taxonomic levels, or by shotgun metagenomics, which is hindered by its high costs and complexity. In this cross-sectional study involving 114 Colombian adults, we used an amplicon-based sequencing strategy with alternative markers—gyrase subunit B (*gyrB*) and DNA K chaperone heat protein 70 (*dnaK*)—that evolve faster than the 16S rRNA gene. Comparing the diversity and abundance observed with the three markers in our cohort, we found a reduction in the diversity of *Clostridia*, particularly within *Lachnospiraceae* and *Oscillospiraceae* among obese individuals [as measured by the body mass index (BMI)]. Within *Lachnospiraceae*, the diversity of *Ruminococcus_A* negatively correlated with BMI. Within *Oscillospiraceae*, the genera *CAG-170* and *Vescimonas* also exhibited this negative correlation. In addition, the abundance of *Vescimonas* was negatively correlated with BMI. Leveraging shotgun metagenomic data, we conducted a phylogenetic and genomic characterization of 120 metagenome-assembled genomes from *Vescimonas* obtained from a larger sample of the same cohort. We identified 17 of the 72 reported species. The functional annotation of these genomes showed the presence of multiple carbohydrate-active enzymes, particularly glycosyl transferases and glycoside hydrolases, suggesting potential beneficial roles in fiber degradation, carbohydrate metabolism, and butyrate production.

**IMPORTANCE:**

The gut microbiota is diverse across various taxonomic levels. At the intra-species level, it comprises multiple strains, some of which may be host-specific. However, our understanding of fine-grained diversity has been hindered by the use of the conserved 16S rRNA gene. While shotgun metagenomics offers higher resolution, it remains costly, may fail to identify specific microbes in complex samples, and requires extensive computational resources and expertise. To address this, we employed a simple and cost-effective analysis of alternative genetic markers to explore diversity within *Clostridia*, a crucial group within the human gut microbiota whose diversity may be underestimated. We found high intra-species diversity for certain groups and associations with obesity. Notably, we identified *Vescimonas*, an understudied group. Making use of metagenomic data, we inferred functionality, uncovering potential beneficial roles in dietary fiber and carbohydrate degradation, as well as in short-chain fatty acid production.

## INTRODUCTION

Bacteria of the class *Clostridia* [phylum *Bacillota A*, formerly *Firmicutes A* (1)] are prominent and diverse members of the human gut microbiota (2–4). Their importance for human health stems from their capacity to break down dietary fiber, produce short-chain fatty acids (SCFAs (*e.g*., butyrate)], and colonize mucins of the gut epithelium (5, 6). Prior studies have indicated that *Clostridia* can constitute up to 40% of the overall microbial diversity in the human gut (7), particularly in non-Western populations (8, 9).

Currently, the class *Clostridia* encompasses many families: 187 according to Genome Taxonomy Database (GTDB) release 08-RS214 (accessed on 13 June 2023). Of these, *Lachnospiraceae*, *Oscillospiraceae*, *Acutalibacteraceae*, and *Ruminococcaceae* stand out as some of the most abundant, prevalent, and diverse families, collectively constituting 58% of all *Clostridia* species. The presence of this class in the human gut has been associated with the regulation of host immunity (10) and its links with host health: a low abundance of *Clostridia* has been associated with type 2 diabetes, obesity, cardiometabolic dysregulation, liver cirrhosis, and Crohn’s disease (6, 11–14). A detailed characterization of the genetic diversity in this group may help to better understand correlations with health parameters and, ultimately, identify species and strains for future development of treatments, such as probiotics.

Molecular approaches have played a crucial role in characterizing microbial diversity, with 16S rRNA gene amplicon sequencing being the most widely used (15, 16). However, this marker evolves at a relatively slow rate, prompting the exploration of less conserved, single-copy housekeeping genes with better phylogenetic signals within a lineage of interest and between closely related clades. For example, Caro-Quintero and Ochman (17) used the gene *gyrB* to reveal hidden diversity in *Bacteroidaceae* and *Lachnospiraceae*. Moeller et al. (18) used the same marker to demonstrate co-speciation between *Bacteroidaceae* and *Biﬁdobacteriaceae* with hominids. La Reau et al. (19) used the *recA* gene to help resolve the intricate phylogenetic relationships within the genus *Ruminococcus* and demonstrated that it has largely unexplored diversity. Guo et al. (20) compared the variation of two single-copy ribosomal protein genes, *rplB* and *rpsC*, with the 16S rRNA and demonstrated that the alternative markers showed more variation than did the 16S rRNA gene in the same organisms and better reﬂected the ecology of microbial communities.

An alternative approach to characterize microbial diversity is the use of shotgun metagenomics [*e.g.*, reference (21)]. However, this method remains expensive and demands substantial computing resources and expertise. Moreover, it typically offers lower sequencing depth for a given taxonomically informative marker compared to an amplicon-based strategy, which is critical for assessing diversity and enrichment. In addition, it presents challenges in evaluating the diversity of lower taxonomic levels in complex biological samples at shallow sequencing depths. Nonetheless, existing shotgun metagenomics data can be easily incorporated and contrasted with the alternative genetic markers, as housekeeping genes are more accurately assembled than the 16S rRNA gene (22).

In this study, our objective was to characterize the diversity of the human gut microbiota belonging to some of the most prevalent, diverse, and abundant families within the class *Clostridia* by using an amplicon-based sequencing strategy with alternative genetic markers that evolve faster than the 16S rRNA gene. We asked whether the diversity within *Clostridia* is greater than previously estimated and, if so, whether the uncovered diversity offered a better resolution of microbiome–host phenotype associations, particularly with human obesity.

## RESULTS

### Genetic variability of the alternative markers and taxonomic coverage of the employed primers

**Fig 1:**
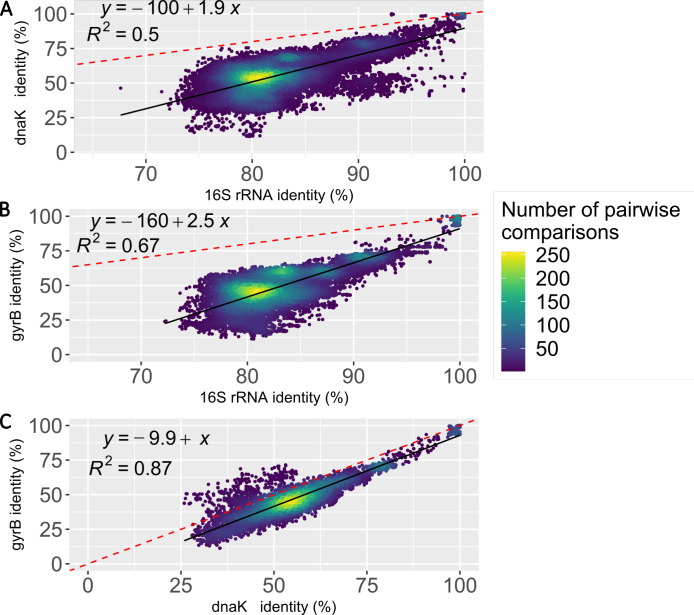
Analysis of the three markers in *Clostridia* genome assemblies. Association of pairwise comparisons among *Clostridia* genome assemblies using 16S rRNA, *dnaK*, and *gyrB* genes. Each dot on the plots represents the pairwise distances of a given pair of assembled genomes: *dnaK* vs 16S rRNA (**A**), *gyrB* vs 16S rRNA (**B**), and *gyrB* vs *dnaK* (**C**). The colors indicate the density of pairwise comparisons. The equation and coefficient are shown for the regression line (continuous black) and the case in which *y* = *x* (red dashed line). Data below the *y* = *x* line indicate that the gene on the *X*-axis is more conserved than the gene on the *Y*-axis. Note that a 16S rRNA sequence identity value of 97%, which is conventionally used to delineate bacterial species, corresponds to 84% identity for *dnaK* and 82.5% for *gyrB*.

In the present study, we used two genetic markers alternative to the 16S rRNA gene: the gene gyrase subunit B (*gyrB*) and the gene DNA K chaperone heat protein 70 (*dnaK*). The primers for *gyrB* were previously published (17), while the primers for *dnaK* were designed by us. We first confirmed that the two alternative markers are more variable in their genetic sequences than the 16S rRNA gene. For this, we retrieved the complete sequences of the three markers from 4,073 public genome assemblies of *Clostridia* and contrasted the level of conservation of these genes by comparing the sequence identities between pairs of taxa. We found that, within the *Clostridia* class, the housekeeping genes *dnaK* and *gyrB* have a broader range of sequence identities (range 25%–100%) in most genome pairs, while 16S rRNA gene identities (range 70%–100%) were more conserved than both *dnaK* and *gyrB* (Fig. 1). We extended the same comparisons at different taxonomic levels (genus, family, and order) and found similar results (Fig. S1). We also conﬁrmed that both alternative markers are present as single-copy in more than 99% of the *Clostridia* genome assemblies.

We then assessed *in silico* the taxonomic coverage of specific primers that amplify fragments of *dnaK* and *gyrB* on 4,270 species representative genomes of the Unified Human Gastrointestinal Genome (UHGG) (8). Primers for *dnaK* amplified 94% and 85% of species within families *Oscillospiraceae* and *Ruminococcaceae*, respectively; they also amplified 55% of species within *Acutalibacteraceae*. These three families belong to the highly diverse, prevalent, and abundant order of *Oscillospirales* and account for 86% of species within it. The *gyrB* primers amplified 40% of species within *Lachnospiraceae*, a family that makes up 96% of the diversity within the order *Lachnospirales* (Table 1; Table S1).

**TABLE 1:** Taxonomic coverage of the employed primers for *dnaK* and *gyrB* within the class *Clostridia*^*a*^

			dnaK		gyrB	
**o__Order**	**f__Family**	**UHGG species representative genomes**	**Primer hits**	**Taxonomic coverage**	**Primer hits**	**Taxonomic coverage**
*o__Oscillospirales*	*f__Oscillospiraceae*	234	221	0.944	23	0.098
*o__Oscillospirales*	*f__Ruminococcaceae*	169	144	0.852	23	0.136
*o__Oscillospirales*	*f__Acutalibacteraceae*	219	121	0.553	41	0.187
*o__Christensenellales*	*f__CAG-74*	36	29	0.806	7	0.194
*o__Oscillospirales*	*f__Butyricicoccaceae*	23	19	0.826	4	0.174
*o__Clostridiales*	*f__Clostridiaceae*	45	18	0.400	5	0.111
*o__Oscillospirales*	*f__CAG-272*	68	17	0.250	14	0.206
*o__Christensenellales*	*f__Christensenellaceae*	12	9	0.750	1	0.083
*o__Christensenellales*	*f__CAG-138*	25	7	0.280	4	0.160
*o__Oscillospirales*	*f__UBA644*	5	5	1.000	0	0
*o__Lachnospirales*	*f__Lachnospiraceae*	470	2	0.004	188	0.400
*o__Christensenellales*	*f__QAND01*	4	2	0.500	0	0
*o__Christensenellales*	*f__UBA1750*	2	2	1.000	0	0
*o__Oscillospirales*	*f__CAG-382*	14	1	0.071	2	0.143
*o__Christensenellales*	*f__GCA-900066905*	1	1	1.000	0	0
*o__Oscillospirales*	*f__QAMX01*	3	1	0.333	0	0
*o__Oscillospirales*	*f__UBA644_A*	4	1	0.250	1	0.250
*o__Peptostreptococcales*	*f__Anaerovoracaceae*	47	0	0	6	0.128
*o__Christensenellales*	*f__QALW01*	9	0	0	2	0.222
*o__Lachnospirales*	*f__Anaerotignaceae*	8	0	0	1	0.125
*o__Lachnospirales*	*f__Cellulosilyticaceae*	3	0	0	1	0.333
*o__Peptostreptococcales*	*f__Peptostreptococcaceae*	25	0	0	1	0.040
*o__Lachnospirales*	*f__UBA1390*	4	0	0	1	0.250

^*a*^ The taxonomic ranks are highlighted as o__ (order) and f__ (family).

### Alpha diversity within *Clostridia* is associated with human obesity

**Fig 2:**
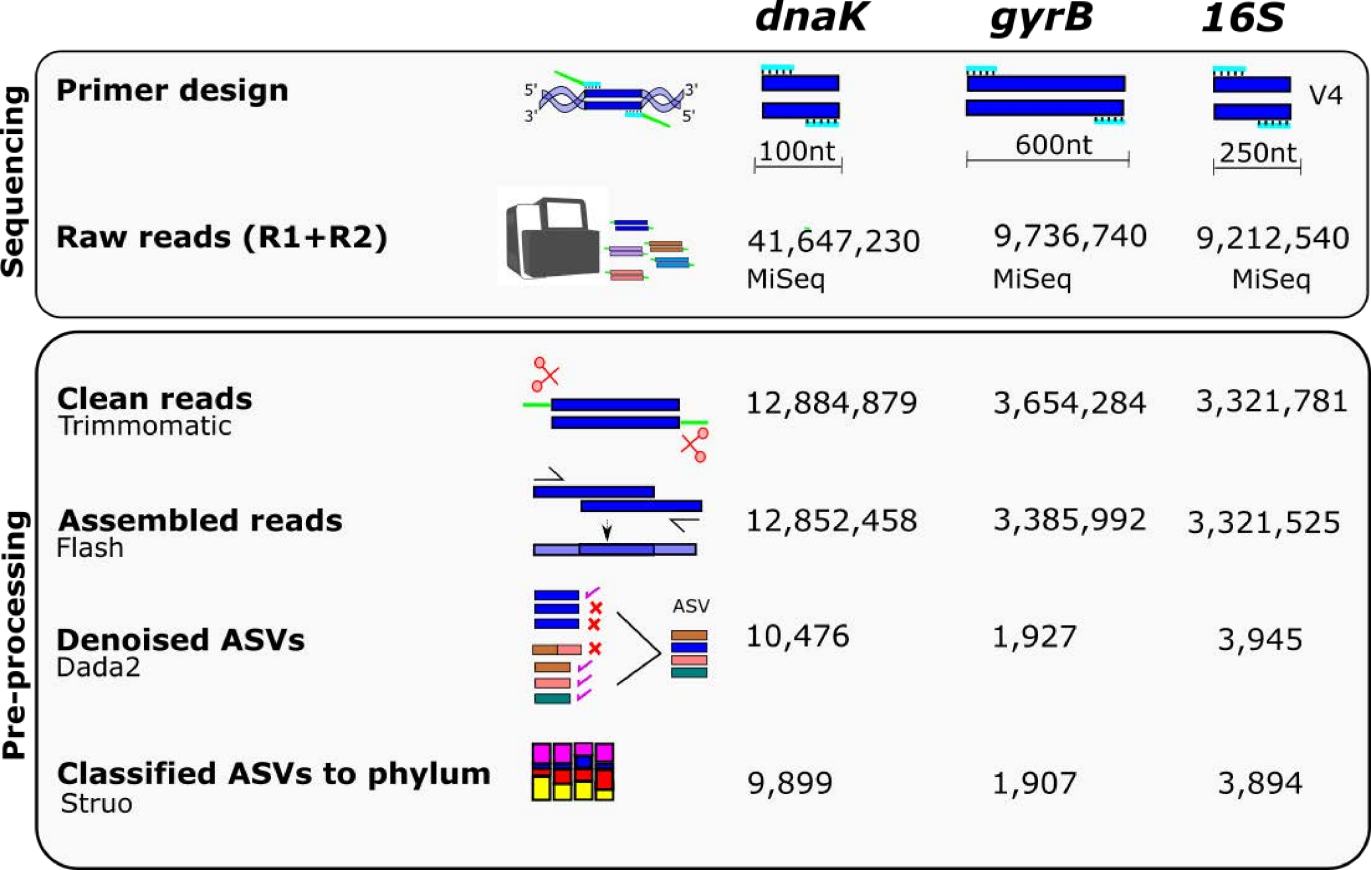
Summary of the methods. Specific primers were used to amplify and sequence fragments of the genes 16S rRNA (hypervariable region V4), *dnaK*, and *gyrB* using the Illumina MiSeq sequencing platform. Trimming of primers was performed using Trimmomatic; complementary reads were assembled using Flash and subsequently denoised using DADA2. ASVs were defined at 100% identity. Taxonomic classification of the amplicons was assigned using the Struo pipeline (24). The numbers of reads and ASVs after each step are shown for each marker.

**Fig 3:**
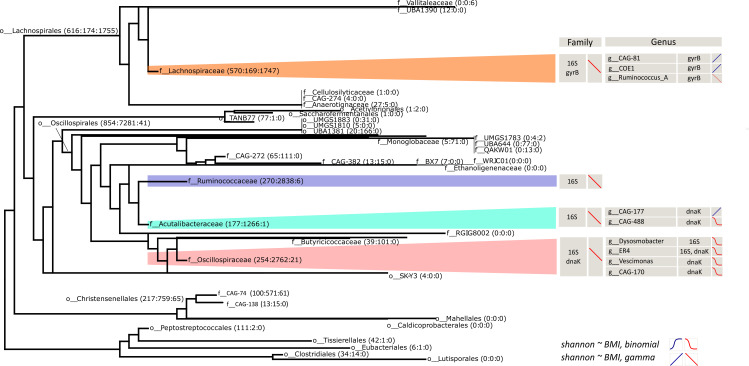
Simplified cladogram of the *Clostridia* class (adapted of a consensus tree from GTDB). The families focused on this study are highlighted (*Lachnospiraceae*, *Ruminococcaceae*, *Acutalibacteraceae*, and *Oscillospiraceae*). In parentheses are the number of ASVs in each group in the order 16S rRNA:*dnaK:gyrB*. The table at the right shows groups whose alpha diversity significantly correlated with body mass index (BMI) according to a binary (glm.bin, sigmoid curve) or continuous (glm.cont, straight line) model and the direction of change (positive in blue, negative in red). The analysis is shown at the family level and for some significant genera.

Next, we examined the diversity of the previously mentioned families in human fecal samples obtained from adult men and women of a well-phenotyped cohort from Colombia (South America), rich in *Clostridia* and studied by us in the past (9, 23). We assessed alpha diversity with the two alternative markers and compared it with 16S rRNA. The *dnaK* and the 16S rRNA libraries were successfully sequenced in the 114 participants (*dnaK*: mean number of reads = 111,761, range = 4,719–202,309; 16S rRNA: mean number of reads = 28,906, range = 3,490–91,366). The *gyrB* libraries were attempted in the same participants but successfully sequenced in 82 of them (mean number of reads = 41,182, range = 10,418–92,539) (Table S2). Ampliﬁed sequences for each marker were grouped into amplicon sequence variants (ASVs) and assigned a taxonomic classiﬁcation (Fig. 2). As expected by their taxonomic coverage, nearly all ASVs detected with the *gyrB* primers belonged to the family *Lachnospiraceae* (1,747 of 1,907, or 92%). In the case of *dnaK*, 69% of the detected ASVs (9,899 total) were largely distributed in three families: *Ruminococcaceae* (2,838, 29%), *Oscillospiraceae* (2,762, 28%), and *Acutalibacteraceae* (1,266, 13%). Of the remaining 3,033 classified ASVs, 1,571 belonged to other families within *Clostridia* (including 759 from the order *Christensenellales,* 8%) and 1,461 outside. Importantly, all these families showed an increased level of detection in ASVs compared to 16S rRNA amplicon sequencing (Fig. 3), which can be interpreted as larger alpha diversity observed using less conserved genes.

**Fig 4:**
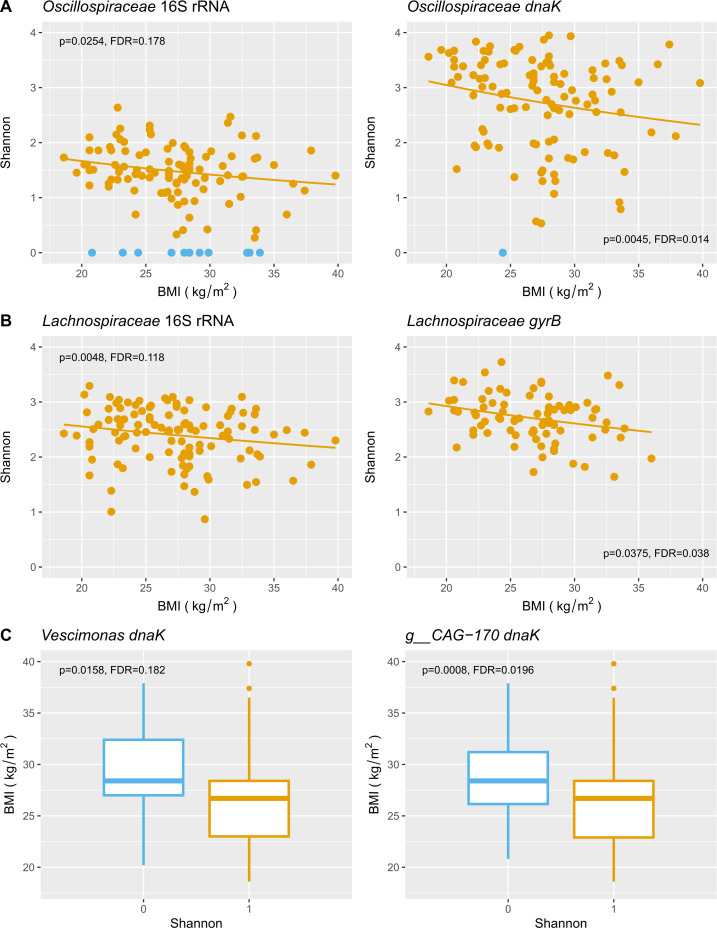
Associations between alpha diversity and BMI for families *Oscillospiraceae* (**A**), *Lachnospiraceae* (**B**), and relevant genera (**C**) of *Clostridia* that display diversity–BMI correlations under two GLM models (continuous model, panels A and B; binary model, panel C). Values of Shannon > 0 in yellow; Shannon = 0 in blue. The corresponding correlation of 16S rRNA for the family is included (panels A and B). *P*-values and FDR-adjusted *P*-values are provided.

Subsequently, we investigated whether the diversity of *Clostridia* in the gut microbiota exhibited any correlation with host health. To explore this, we assessed the association with obesity, as measured by the BMI, for the amplified families and all the genera within these families. This analysis was conducted using two types of generalized linear models (GLMs; see Materials and Methods). These models included the participant’s city of origin as a covariate to account for differences in the baseline diversity. Including other covariates in our models (e.g., sex, age, stool consistency, and medicament consumption) yielded similar results. We found signiﬁcant, negative correlations between alpha diversity and BMI using continuous GLMs for the family *Oscillospiraceae* with both *dnaK* [glm.cont: beta = −0.017, *P* = 0.0045; false discovery rate (FDR) = 0.014] and 16S rRNA (glm.cont: beta = −0.014, *P* = 0.0254; FDR = 0.178), and for the family *Lachnospiraceae* with both *gyrB* (glm.cont: beta = −0.010, *P* = 0.0375, FDR = 0.038) and 16S rRNA (glm.cont: beta = −0.011, *P* = 0.0048; FDR = 0.118) (Fig. 4A and B). Within *Lachnospiraceae*, the genera (*g__* denotes genus) *g__CAG-81* (glm.cont: beta = 0.202, *P* = 0.0137; FDR = 0.157) and *g__COE1* (glm.cont: beta = 0.386, *P* = 0.009; FDR = 0.157) showed significant positive correlations with BMI, while the genus *Ruminococcus_A* a negative one (glm.cont: beta = −0.073, *P* = 0.019; FDR = 0.157) (Fig. 3). The signiﬁcant genera within the *Oscillospiraceae* family, *g__CAG-170* (glm.bin: beta = −0.165, *P* = 0.0008; FDR = 0.0196) and *Vescimonas* (glm.bin: beta = −0.11, *P* = 0.0158; FDR = 0.182) were signiﬁcant under the binary model (Fig. 3 and 4C), suggesting a threshold effect, where the probability of having a high diversity sharply decreases above certain BMI values (i.e., with obesity). The families *Ruminococcaceae* (glm.cont: beta = −0.013, *P* = 0.039; FDR = 0.231) and *Acutalibacteraceae* (glm.cont: beta = −0.032, *P* = 0.0040; FDR = 0.118) showed negative correlations with BMI when evaluated with the 16S rRNA gene. Within *Acutalibacteraceae*, *g__CAG-177* exhibited a positive correlation with BMI (glm.cont: beta = 0.062, *P* = 0.0254; FDR = 0.127). Other families and genera that were associated with BMI are shown in Fig. 3; Table S3. A sensitivity analysis indicated that the above associations were largely robust to clustering at 97% sequence identity, but as expected, signiﬁcance decreased with clustering at lower identities (Table S4).

We tested the generalizability of the above results by analyzing the mentioned families and genera in a set of global metagenomes including 1,153 samples from 10 populations (seven Westernized and three non-Westernized) (25). We confirmed the aforementioned negative associations between microbial alpha diversity and BMI using continuous GLMs for families *Oscillospiraceae* (glm.cont: beta = −0.004, *P* = 0.013; FDR = 0.053), *Acutalibacteraceae* (glm.cont: beta = −0.005, *P* = 0.03; FDR = 0.056), and *Lachnospiraceae* (glm.cont: beta = −0.002, *P* = 0.064; FDR = 0.085), although effect sizes were four to six times weaker than in the Colombian cohort (Table S3). For the genera, we confirmed a positive correlation between BMI and alpha diversity using the continuous GLM for *g__CAG-81* (glm.cont: beta = 0.005, *P* = 0.008) and negative correlations within *g__CAG-170* (glm.cont: beta = −0.007, *P* = 0.01) and *Vescimonas* (glm.cont: beta = −0.006, *P* = 0.035), again, with weaker effects. *Vescimonas* also showed a negative correlation with BMI using the binary model (glm.bin: beta = −0.059, *P* = 0.03); in this case, the effect was half that in the Colombian population (Table S3). Note that we did not adjust *P*-values for the mentioned genera as we did not exhaustively test all of them in the global metagenomes. This data set was only intended to assess the potential generalizability of our results.

Taken together, our results indicate that lean individuals (*i.e.*, low BMI) harbor a greater diversity of key *Clostridia* members than obese subjects (*i.e.*, high BMI) and that using more variable genetic markers allowed us to identify associations with a host phenotype that seem to be partly shared by different populations.

### *Clostridia* abundance is associated with human obesity

**Fig 5:**
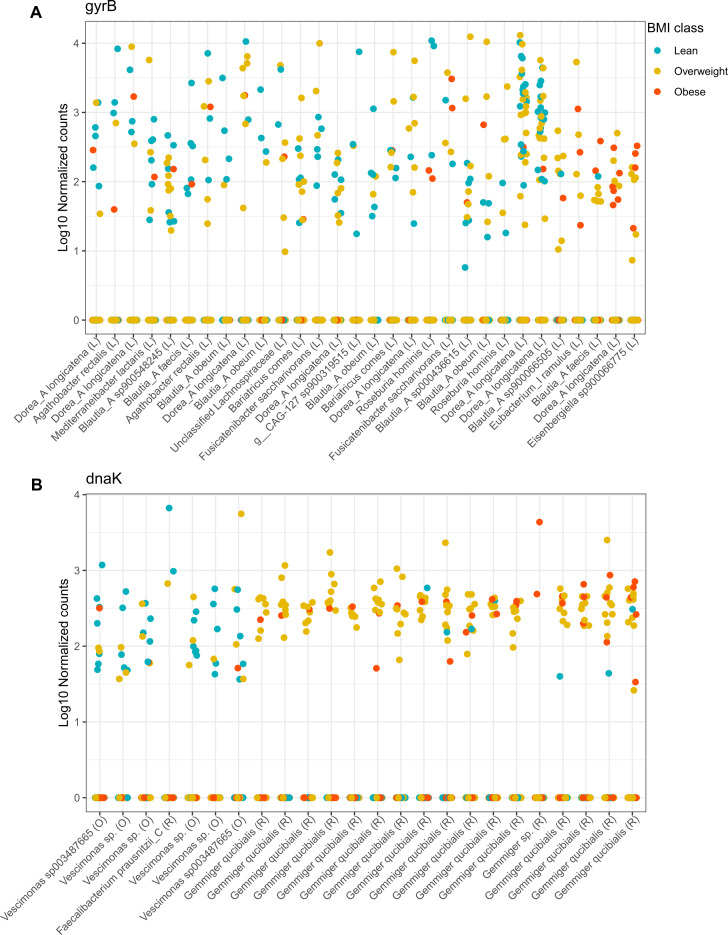
Associations between bacterial abundance and BMI. ASVs with differential abundance according to the host phenotype (lean, blue; overweight, yellow; obese, red). The (log 10) normalized counts of each ASV in each sample are shown. (**A**) ASVs detected with *gyrB*. (**B**) ASVs detected with *dnaK*. The family each ASV belongs to is indicated in parenthesis (O, *Oscillospiraceae*; R, *Ruminococcaceae*; L, *Lachnospiraceae*).

Next, we evaluated the association of the abundance of *Clostridia* members with BMI using the negative binomial GLM implemented in DESeq2 (26). We tested differential abundance between lean and obese individuals for all ASVs. No ASV within *Clostridia* differed significantly in abundance according to 16S rRNA gene data. In contrast, 30 ASVs for *gyrB* belonging to 14 species were signiﬁcant, and for *dnaK* 24 ASVs, belonging to ﬁve species (Fig. 5). The significant ASVs with *gyrB* did not show consistency in the direction of over- or underrepresentation at the taxonomic level (Fig. 5A). An example of this is the species *Dorea_A longicatena*, which shows abundance enrichment for two ASVs in lean and overweight subjects, while nearly absent in obese individuals. Yet, another ASV of the same species was enriched in obese and overweight samples while absent in lean individuals. This could be explained by either strain-specific associations with obesity or taxonomic misclassification. The *dnaK* marker, on the other hand, revealed all but one signiﬁcant ASV overrepresented in lean compared to obese individuals belonging to the genus *Vescimonas*, whereas ASVs underrepresented belonged to the genus *Gemmiger* (mainly, *Gemmiger qucibialis*) (Fig. 5B). Results for the contrast lean vs overweight were largely consistent (Table S5).

### Metagenome-based inference of the functional potential of *Vescimonas*

We next focused on a single clade, the genus *Vescimonas*, since it showed a robust association with host phenotype: a negative correlation between BMI and alpha diversity (Fig. 4C), along with an enrichment of several ASVs in lean individuals (Fig. 5B). The fact that *Vescimonas* was only recently isolated (27) further increased our interest in characterizing this group in the context of human obesity. For this, we used a complementary approach, in which we leveraged recently assembled MAGs from the same samples used in the present study to determine the overall characteristics of this clade that might explain its association with host health (28). We used a set of 120 MAGs classified as *Vescimonas*, together with 71 publicly available genomes corresponding to species representatives from GTDB (Table S6), in total 193 MAGs. We complemented this analysis with the annotated genomes of *Vescimonas coprocola*, type strain MM50, and *Vescimonas fastidiosa*, strain MM35 (27).

**Fig 6:**
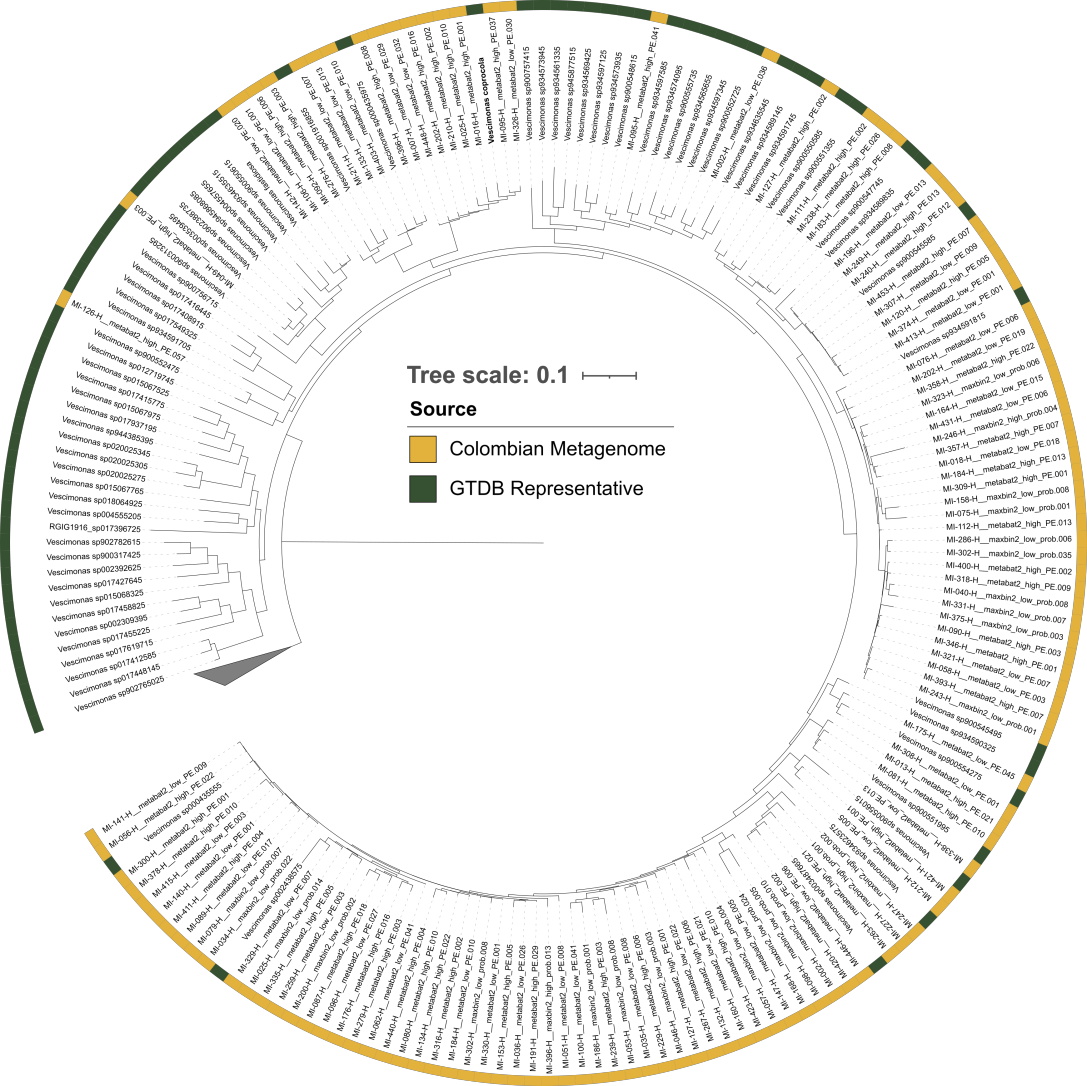
Phylogeny of *Vescimonas*. Maximum-likelihood phylogeny of *Vescimonas* with the 120 MAGs assembled from Colombian samples (yellow ring) and 73 representative genomes from the GTDB (gray ring). The scale bar represents the number of substitutions per site.

We first performed a phylogenetic and taxonomic classification of this set of MAGs. We observed that the 120 MAGs assembled from Colombian subjects were closely related to existing GTDB species representatives, mainly the uncultured *Vescimonas* sp000435555 (Fig. 6; Fig. S2). We detected 17 out of the 27 reported *Vescimonas* species in the MAGs from Colombians; conversely, 20 *Vescimonas* species could be identified at the species level with the *dnaK* marker while only one with the 16S rRNA gene. Although the number of species found with the different methods may not be directly comparable, we inspected the diversity and abundance of the *Vescimonas* MAGs in relation to BMI. We found a similar trend as with the amplicon-based approach: a lower probability of *Vescimonas* diversity above a certain BMI value (glm.bin, *P* = 0.106; Fig. S3) and higher, albeit not significant counts of certain *Vescimonas* species in lean individuals with respect to obese (Fig. S4).

**Fig 7:**
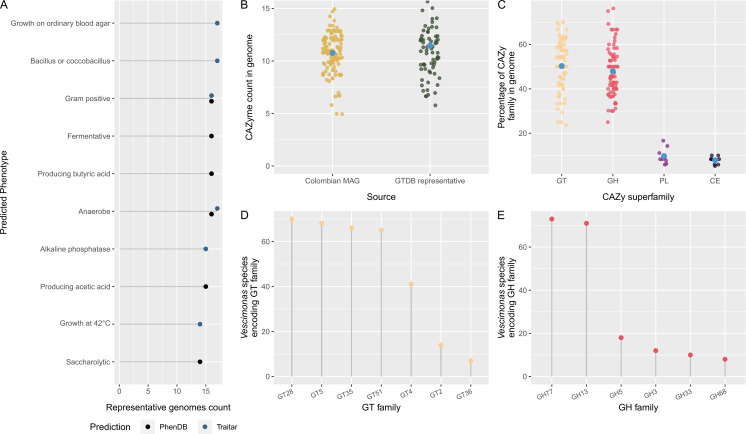
Functional annotation of *Vescimonas*. MAGs were assembled from Colombian subjects (120 genomes corresponding to 17 species) and GTDB species representatives (73 genomes). (**A**) The number of *Vescimonas* species detected in the Colombian data set with a given trait as predicted by Traitar (black circles) and PhenDB (blue circles). (**B**) Total number of carbohydrate-active enzymes (CAZymes) in Colombian MAGs and GTDB representatives; blue dots represent mean values across all MAGs. (**C**) Average percentage of the total number of CAZymes belonging to each CAZy superfamily per species (GT, glycosyl transferases; GH, glycoside hydrolases; PL, polysaccharide lyases; CE, carbohydrate esterases); blue dots represent values across all species. (**D** and **E**) Most frequently detected GT (**D**) and GH (**E**) CAZymes in *Vescimonas*; the plot indicates the number of distinct *Vescimonas* species with at least one genome in which the enzyme was detected in the combined Colombian and GTDB genome set.

Next, we used the genomes of the species representatives detected in the Colombian metagenomes to gain insights into the phenotypic characteristics of members of *Vescimonas*. For this, we predicted a series of microbial traits using Traitar (29) and PhenDB (30). We considered a trait to be robustly predicted if it was reported in at least 2/3 of the genomes. The prediction results suggest that members of *Vescimonas* are bacillus or coccobacillus-shaped Gram-positive anaerobes. They are also predicted to be phosphatase alkaline-positive and able to grow on ordinary blood agar at a temperature of 42°C. In addition, these bacteria are predicted to have a fermentative, saccharolytic lifestyle and can produce acetic and butyric acids (Fig. 7A). We contrasted these predictions with the previously reported *in vitro* characterization of *Vescimonas coprocola*-type strain MM50 (27). In agreement with genome-based predictions, strain MM50 is an obligate anaerobic, non-motile, non-spore-forming bacillus. It grows in an ordinary medium (GAM) incubated at 25°C–40°C and exhibits (weak) alkaline phosphatase activity. Its major end product is n-butyrate. In contrast with our predictions, it stained Gram-negative (27), which is unexpected for *Clostridia*.

To gain insights into the functional potential of *Vescimonas* in the human gut that might explain its association with host health, we performed gene calling on all the GTDB species representatives and Colombian MAGs using Prokka (31) and annotated the predicted proteins using eggNOG mapper (32). We first sought to determine whether members of *Vescimonas* can ferment plant fibers and contribute to human leanness through a healthy diet. For this, we assessed the repertoire of CAZymes in the analyzed genomes. We compared the distribution of CAZymes in the Colombian MAGs and the GTDB representatives and did not ﬁnd differences in the total CAZyme count per genome (Welch’s *t*_38.65_ = 0.078, *P* = 0.90) (Fig. 7B). We then assessed individual CAZy superfamilies and found that glycosyl transferases (GTs) and glycoside hydrolases (GHs) were the most frequent CAZymes in *Vescimonas* genomes, representing 52.86% ± 6.81 and 46.74% ± 6.76 of the carbohydrate-active enzymes detected in a given accession, respectively (Fig. 7C). Among GTs, the most widespread enzymes were GT35, a starch phosphorylase; GT5, a starch glucosyl transferase; GT28, a 1,2-diacylglycerol 3-beta-galactosyl transferase, and GT51, a murein polymerase (Fig. 7D). In the case of GHs, the most prevalent enzymes in *Vescimonas* species were labeled as GH13 and GH77, enzymes with alpha-amylase activity (Fig. 7E). In agreement with this, *V. coprocola* MM50 seems also able to ferment dietary fiber, as it has genes that map onto the CAZy superfamilies GT2, GT5, GT28, GT35, and GT51, and GH13, GH25, and GH77 (Table S7).

## DISCUSSION

In the present study, we characterized the diversity of the bacterial class *Clostridia* using markers other than the 16S rRNA gene, thus obtaining a finer taxonomic resolution. Our results showed that the 16S rRNA gene underestimated the diversity of *Clostridia* due to the conservation of its sequence and that faster-evolving marker genes may be more suitable to assess the diversity at intra-species taxonomic levels. We further correlated the diversity of this clade we observed in a set of 114 human adults with host obesity. Given the disparities in simple associations of genus and species with health and disease (3, 21, 33, 34), looking into the link between host health and strain-level diversity becomes highly relevant.

We used an amplicon-based strategy to gain resolution in the characterization of diversity within key *Clostridia* families. The advantage of this strategy over a metagenomic-based search when studying specific clades is that it produces better depth to assess differences in abundance and richness, facilitating an improved characterization of microbiome diversity and host–microbe interactions, as shown by Youngblut et al. (35). A search in the metagenomic data set we assembled with the same fecal samples showed that only a minor proportion (~0.1%) of the annotated genes were labeled as *gyrB* and *dnaK*. Additionally, the molecular protocols for amplicon sequencing are simpler and more affordable and, therefore, more easily replicated. In this study, we focused on the *Clostridia* class, one of the dominant groups of the human gut microbiota associated with human health (2–6). However, this methodological approach can easily extend to other populations and groups of microbes, for instance, those displaying signatures of health and disease (14, 36, 37).

We showed that the diversity of *Clostridia*, particularly within the families *Lachnospiraceae*, *Oscillospiraceae*, and *Acutalibacteraceae,* was correlated with host health. Interestingly*,* we observed extensive heterogeneity in the ASV abundance distribution in the studied population. Most ASVs were present only in a subset of samples, and only a few ASVs were present in many samples. Consequently, the ASVs that were found significantly differentially abundant with the host phenotype (Fig. 5) were few and among the most prevalent (Fig. S5), as those had higher statistical power to be detected. Intra-species heterogeneity has been associated with diet, among other host factors (38).

We further found that the richness of the genus *Vescimonas* (*Oscillospiraceae*) and the abundance of multiple ASVs classified as such showed a negative correlation with BMI. This is noteworthy because this consistent association was observed across different taxonomic levels and could be used as a potential marker of gut health. *Vescimonas* is a poorly studied member of the gut microbiome. Formerly known as *g__CAG-83* (GTDB v214), it comprises two species cultured so far, *V. coprocola* MM50 and *V. fastidiosa* MM35 (27). This genus has been associated with leanness (39) and has gained attention as a potential probiotic (40). Indeed, the results of our analysis of the genomes of the sequenced strains and the MAGs from our cohort further indicate that members of this clade are carbohydrate-utilizing, butyrate-producing bacteria. This together with the aforementioned association of *Vescimonas* ASVs with BMI hints at an important role for the human host. A deep characterization is needed for this group, which should include additional efforts in culturing, sequencing, and annotating speciﬁc strains.

The abundance of other groups was associated with host health. *Gemmiger qucibialis* (*Ruminococcaceae*) was more abundant in obese than lean subjects. *Gemmiger* is a sister clade of *Faecalibacterium*; it is phylogenetically close to *Ruminococcus* and constitutes a clade that can be very abundant but has been neglected in human microbiome research (3). Pasolli et al. (3) highlighted the divergence of multiple strains of *Cibiobacter qucibialis*, showing differences between non-Westernized and Westernized populations. Our results support the existence of multiple strains within *G. qucibialis*, several of which would be associated with obesity. In contrast, *Faecalibacterium prausnitzii* clade C was associated with leanness. *Faecalibacterium prausnitzii* is one of the most prevalent species in the human gut (4), and its abundance is considered a marker of gut health (41). De Filippis et al. (33) demonstrated that *Faecalibacterium* is a complex of species that comprises 11 clades. Clade C showed geographic distinction and was found to have a higher prevalence in non-Western compared to Western populations. Consistently, the microbiota of our studied population (not included in the mentioned publication) corresponds to an urban, non-Western population, in a transition to a Westernized lifestyle (9). Other species that showed higher abundance in lean than obese individuals were *Agathobacter rectalis*, *Roseburia hominis*, *Bariatricus comes*, and *Fusicantenibacter saccharivorans*, which have been reported to be abundant components of the core microbiome of healthy humans (4, 42, 43). ASVs belonging to *Dorea_A* and *Blautia_A* had a dual response concerning BMI. Both *Blautia* and *Dorea* have been found enriched in individuals with excess weight (43), but our results suggest that this correlation may be particular to some ASVs (*e.g.*, strains) and not the genus as a whole.

Our study has several strengths: the methodology proposed here with alternative markers improves the resolution of intra-species diversity within specific clades of the Tree of Life. Additionally, it is more straightforward, affordable, and easily replicated compared to a whole genome metagenome-based approach. However, we also recognize certain limitations. The need to design speciﬁc primers for the alternative marker genes and the dependence on taxonomic databases to annotate ASVs may pose a challenge. Furthermore, the designed primers may not fully cover diverse groups of microbes, as was our case with *gyrB* for the family *Lachnospiraceae* since they missed a considerable part of the diversity within this family (~60%). We were able to partly reproduce the associations of diversity and obesity in a set of global metagenomes, although with weaker effect sizes. While these results support the general observation that a loss in diversity in *Clostridia* is associated with detrimental health (11–13) and alterations in the composition of the gut microbiome (14), it remains to be determined the population-specific features that contribute to stronger or weaker associations between taxon-specific alpha diversity and BMI.

In conclusion, our amplicon-based approach on faster-evolving genes than the 16S rRNA gene was useful in revealing some of the hidden diversity in the human gut microbiota while remaining cheaper, simpler than a shotgun metagenomics-based search, and more powerful for analyzing specific groups of microbes. Our study case in the *Clostridia* class allowed us to deeply assess this phylogenetic group’s diversity and reveal meaningful associations with host health.

## MATERIALS AND METHODS

In this article, we followed the reporting guidelines proposed by the “Strengthening The Organization and Reporting of Microbiome Studies” (STORMS) consortium (44). The STORMS checklist is provided in Table S8.

### Samples

Between July and November 2014, 459 male and female adults between 18 and 62 years of age from the general population were recruited in approximately equal proportions according to BMI categories (lean, overweight, and obese), the city of residence (Bogota, Medellin, Cali, Barranquilla, or Bucaramanga, all located in Colombia, South America), sex at birth (males, females), and age range (18–40 and 41–62 years). The weight and height of participants were measured by a trained research team member using Cardinal Detecto DR400C digital scales (St. Webb City, MO, USA) and Seca portable measuring rods (Hamburg, Germany). BMI was calculated as weight (kg)/height squared (m^2^) to classify participants as lean (18.5 ≤ BMI < 25.0 kg/m^2^), overweight (25.0 ≤ BMI < 30.0 kg/m^2^), or obese (BMI ≥ 30.0 kg/m^2^). All included participants were insured by the health insurance provider EPS Sura. We excluded underweight individuals (BMI < 18.5 kg/m^2^), pregnant women, individuals who had consumed antibiotics or antiparasitics less than 3 months before enrolment, and individuals diagnosed with Alzheimer’s disease, Parkinson’s disease, or any other neurodegenerative diseases; current or recent cancer (<1 year); and gastrointestinal diseases (Crohn’s disease, ulcerative colitis, short bowel syndrome, diverticulosis, or celiac disease). Written informed consent was obtained from all the participants before beginning the study.

Gut microbiota characterization via 16S rRNA gene sequencing was completed in 441 participants, as previously described (9). The present study used a subset of 114 samples of individuals from the aforementioned cross-sectional study (Table S2). Fecal samples were immediately refrigerated in household freezers and brought to a local facility within 12 hours, where they were stored in dry ice and sent to a central laboratory via next-day delivery. Samples were aliquoted and kept at −80°C until DNA extraction.

### Primer design

Primers for the family *Lachnospiraceae* were previously published (17) and correspond to a fragment of ~600 bp of the gene gyrase subunit B (*gyrB*) (F_La_334–354: GGHGGAGGATAYAAGGTATCC; R_La_816–836: TRTANGAATCRTTRTGCTGC). To design primers for the families *Oscillospiraceae*, *Ruminococcaceae*, and *Acutalibacteraceae*, a comparative analysis of orthologous sequences was carried out using OrthoDB v10 (https://www.orthodb.org). The gene DNA K chaperone heat protein 70 (*dnaK*) was selected as it was present in all species reported and was single-copy, and the conditions for denaturation temperature and %GC were predicted to be similar between forward and reverse primers. The DNA sequences were translated into amino acids, aligned using ClustalW (45), and reverse-translated into nucleotides while keeping the alignment with PAL2NAL (46). These alignments were subsequently analyzed with speciﬁc scripts to automatically search for the primers (17). In this way, a fragment of ~90 bp of *dnaK* was selected and ampliﬁed with custom primers (F_Ru_45_65: YGTNGCNGTNATGGARGGCGG; R_Ru_154_174: NGCCTGNCKYTTTGCVACCTG). The V4 hypervariable region of the 16S rRNA gene from each sample was ampliﬁed using primers F515 (GTGCCAGCMGCCGCGGTAA) and R806 (GGAC TACHVGGGTWTCTAAT), as previously described (9).

### DNA extraction and PCR conditions

Total microbial DNA was extracted using the QIAamp DNA Stool Mini Kit (Qiagen, Hilden, Germany) following the manufacturer’s instructions with a modiﬁcation consisting of a bead-beating step with the lysis buffer (20 sec. at 15 Hz). After extraction, we quantiﬁed the DNA concentration using a Nanodrop spectrophotometer (Nyxor Biotech, Paris, France). DNA extraction was achieved at Vidarium’s laboratory (Medellin, Colombia). For *dnaK* ampliﬁcation, the ﬁnal volume of the reaction was 25 µL, containing 12.5 µL of Taq PCR Master Mix (Qiagen), 1 µL of forward primer (10 mM), 1 µL of reverse primer (10 mM), 1 µL of fecal metagenomic DNA, and 9.5 µL of ultrapure distilled water. The PCR conditions were as follows: initial denaturation at 95°C for 10 min.; 35 cycles of 95°C for 15 sec., 62°C for 30 sec., and 72°C for 20 sec.; and elongation at 72°C for 10 min. For *gyrB*, PCR conditions were as in Caro-Quintero and Ochman (17). The final volume of the reaction was 20 µL, containing 8 µL of 5-Prime HotMasterMix 2.5×, 1 µL (100 ng) of sample DNA, 1 µL (10 mM) of each of the forward and reverse primers containing adaptor sequences, and 9 mL of ultrapure distilled water. The PCR conditions were as follows: initial denaturation at 95°C for 120 sec.; 30 cycles of 95°C for 15 sec., 62°C for 30 sec., and 72°C for 90 sec.; and a final extension at 72°C for 10 min. For 16S rRNA ampliﬁcation, the ﬁnal volume of the reaction was 20 µL, containing 2 µL of 10× AccuPrime PCR Buffer II, 0.15 µL of AccuPrime HiFi Polymerase, 5 µL of the primer set (4 mM), 1 µL of fecal metagenomic DNA, and 11.85 µL of ultrapure distilled water. The PCR conditions were as follows: initial denaturation at 95°C for 2 min.; 30 cycles of 95°C for 20 sec., 55°C for 15 sec., and 72°C for 5 min.; and elongation at 72°C for 10 min.

### Amplicon sequencing

Sequencing was done using Illumina MiSeq technology. The amplicon for the 16S rRNA gene was sequenced using MiSeq platform v2 500 (2 × 250 paired-ends), using a mock community (HM-782D, BEI Resources, Manassas, VA) as a positive control and negative controls (ultrapure water and a DNA extraction blank), as previously described (9). For the sequencing of the *dnaK* and *gyrB* libraries, metagenomic DNA obtained from the participants was sent to the laboratory Applied Biological Materials Inc. (Richmond, BC, Canada) for ampliﬁcation, multiplexing, and sequencing. The *dnaK* libraries were sequenced in Illumina MiSeq cycle ﬂow cell (2 × 150 paired-ends) while the *gyrB* libraries were sequenced in Illumina MiSeq v3 600 (2 × 300 paired-ends). Samples were randomized before library preparation and sequencing to reduce batch effects. Sequencing of the 16S rRNA and *dnaK* libraries was attempted and achieved in 114 participants, and sequencing of the *gyrB* libraries was attempted in 114 participants but only achieved in 82 of them. As analyses were performed separately for each marker, these missing data did not affect the results of the other markers. A summary of the number of reads and ASVs for each marker can be found in Fig. 2.

### Processing of amplicon sequences

Reads were initially pre-processed with Trimmomatic v0.39 (47) using a minimum quality score of 20 and a window size of 4. Adapter removal was performed, and a minimum sequence length was established at 100 bp. Complementary reads were assembled using Flash v1.2.11 (48). For *dnaK* and *gyrB* sequences, the amplified region was purified *in silico*, by extracting the inner sequence between primer pairs using Seqkit amplicon v2.1.0 (49). For 16S rRNA, primers were removed using an in-house Python script. Subsequently, denoising was performed using DADA2 (50) in denoised-single mode, as implemented in Qiime2 v2020.2 (51). A truncation length of 120, 440, and 250 bp was set for the *dnaK*, *gyrB*, and 16S rRNA sequences, respectively. Finally, reads were collapsed into representative sequences, known as ASVs (52) at 100% identity. Taxonomic classiﬁcation of the amplicons was performed using Kraken 2 with default parameters using databases generated with the Struo pipeline (24) (available at http://ftp.tue.mpg.de/ebio/projects/struo2).

### Characterization of *dnaK* and *gyrB* sequences of *Clostridia* in public databases

To build a local database of reference genomes of the class *Clostridia*, a search of genome assemblies was performed through the GTDB. We selected accession numbers of assemblies that belong to the *Clostridia* class according to the GTDB taxonomy, which have high completeness (≥95%) and low contamination (<1%). Using the selected accession numbers, a total of 4,073 assemblies were downloaded on 28 October 2020 from the NCBI. Within the downloaded assemblies, we had both types of data: culture-independent MAGs and genome assemblies from cultured and isolated strains, both with different assembly/fragmentation levels. This set of selected assemblies constituted the local database (makeblastdb version 2.6.0). The query sequences for *dnaK* and *gyrB* were downloaded from the UniProt database. For *dnaK*, we selected a protein sequence classiﬁed as Chaperone protein DnaK (A0A143WYF3), gene *dnaK*, size: 594 amino acids, from the organism *Clostridiales* bacterium CHKCI006. For *gyrB*, we selected a protein sequence classiﬁed as DNA gyrase subunit B (R6D2S9), gene *gyrB*, size: 680 amino acids, from the organism *Ruminococcus* sp. CAG:579. The 16S rRNA query sequence was downloaded from the NCBI and reported as a “16S ribosomal RNA partial sequence” (NR_074399.1), size 1,500 bp, from the source organism *Ruminococcus albus* 7 = DSM 20455. Each query sequence was used to ﬁnd the coordinates of the queried gene on each whole assembly sequence in the local database. For this, we used two BLAST tools provided by the NCBI. For the 16S rRNA gene, we used BLASTn (parameters: -max_target_seqs 5000, -max_hsps 1, -evalue 0). For the protein-coding genes *dnaK* and *gyrB*, we used tBLASTn (parameters: -max_target_seqs 5000, -evalue 0). Although we purposely chose housekeeping genes (*dnaK* and *gyrB*) with one expected copy per genome, we checked for possible occurrences of multi-copies.

For each gene, we carried out an alignment with the retrieved sequences using MAFFT (53). The alignments were visually inspected for quality check. During this process, the *gyrB* alignment was trimmed at the ends keeping the section between 1,281 and 3,955 nucleotide positions. We built pairwise distance matrices from the alignments, by considering each pair of sequences separately to calculate a respective distance value. The pairwise distances give an estimation of the degree of similarity (0) or dissimilarity (1) of two sequences from two different genome assemblies. We calculated the distances between every pair of sequences using APE 5.0 (54) with the Kimura 1980 evolution model (K80).

### Taxonomic coverage analysis

We tested the taxonomic coverage of the *dnaK* and *gyrB* primers described above on 4,270 species representative genomes of the UHGG. UHGG genomes cluster more than 200,000 non-redundant prokaryotic genomes, including MAGs and isolate genomes (8). For this, we performed *in silico* PCRs on each genome using the software ipcress (https://www.ebi.ac.uk/about/vertebrate-genomics/software/ipcress-manual). We considered a range of 75–150 bp for calling a hit with *dnaK* primers and 800–1,100 bp for *gyrB*. We also allowed up to six mismatches for both primers.

### Statistical analysis

Analyses were undertaken in R v.4.3.1 (55) using the package phyloseq v1.44.0 (56) to import the table of counts, tree, taxonomy, and metadata ﬁles. We performed independent analyses for each marker gene (16S rRNA, *dnaK*, and *gyrB*). We excluded samples and ASVs with less than 100 counts in downstream analyses. For alpha diversity analyses, we normalized read counts across samples by rarefying data sets according to the sample with the lowest read number. For the differential abundance analysis, read counts were normalized by sample-speciﬁc size factors determined by the median ratio of ASV counts relative to the geometric mean of all ASVs using the package DESeq2 1.40.2 (26). To test whether an association exists between alpha diversity and BMI, two models were deﬁned with the Shannon index as the response variable and continuous BMI as explanatory: (i) glm.bin: binary (zero vs non-zero), which takes into account overdispersion (zero-inﬂated) of ASVs through a generalized linear model with binomial distribution and logit link, and (ii) glm.cont: continuous for Shannon index larger than zero, through a generalized linear model with Gamma distribution and log link. All the GLM models were adjusted to the participant’s city of origin, as this variable showed differences in the baseline diversity. All significant findings (*P*-values < 0.05) were reported. *P*-values were adjusted for multiple testing using the FDR method and considered significant at FDR < 0.20. For sensitivity analyses, we ran additional GLMs in which sex at birth, age, stool consistency, and medication consumption were included. Also, we collapsed ASVs at 97%, 90%, and 85% identity to check the robustness of our results with the decipher 2.28.0 package of R.

### Analysis of a set of global metagenomes

We downloaded a set of metagenomes from 28 countries, comprising 3,234 samples (25). We trimmed the data set to the 1,153 samples (10 populations: 3 non-Westernized and 7 Westernized) with BMI > 18.5 kg/m^2^, as in the Colombian cohort. We extracted specific taxonomic annotations and abundances, obtained alpha diversity estimates (Shannon index), and performed the same statistical models described above to correlate alpha diversity and BMI. Note that in this data set, we only tested associations between microbial alpha diversity and BMI for the taxa found in the Colombian cohort.

### Metagenome sequencing and assembly

For 430 samples of the original cohort, we performed shotgun metagenome sequencing and obtained MAGs, as described elsewhere (28). Brieﬂy, 1 ng of fecal DNA was used for Nextera Tn5 tagmentation (57). Samples were normalized, pooled, and size-selected. We then performed 2 × 150-bp paired-end sequencing on barcoded pools using the Illumina HiSeq 3000 platform. We removed adapters and low-quality sequencing reads with fqtools v.2.0 (58), bbtools v37.78 (https://jgi.doe.gov/data-and-tools/bbtools/), and skewer v.0.2.2 (59). We ﬁltered human reads *in silico* by mapping them to the hg19 assembly with the “bbmap” command of bbtools. Metagenome coverage was calculated with Nonpareil v.3.3.4 (60); we retained 408 samples with a sequencing depth above 1 million reads or a metagenome coverage over 60% for metagenome assembly. Metagenome assembly was performed using the workﬂow developed by Youngblut et al. (61). Each sample was subsampled to a maximum of 20 million reads. Then, a reference-based metagenome assembly was performed on each separately using MetaCompass v.1.2 (62). We used metaSPAdes v.3.12.0 (63) to perform a *de novo* assembly of reads that did not map to any reference genome. We combined and de-replicated contigs reference-based and *de novo* assemblies for each sample, retaining contigs with at least 2,000 bp. Per sample binning of contigs was performed with MaxBin2 v.2.2.4 and MetaBAT2 v.2.12.1. We selected the best non-redundant set of contig bins (*i.e.*, MAGs) using DAS-Tool v.1.1.1 (64). MAGs from all samples were combined for downstream analyses. Completeness and contamination estimates were calculated for each MAG using CheckM v.1.0.13. MAGs with completeness of <50% or contamination of ≥5% were discarded. Clonal genomes were collapsed at an average nucleotide identity of 99.9% using dRep (65). We assigned the taxonomic classiﬁcation of the mid- and high-quality non-redundant MAGs with GTDB-Tk v.0.3.3 (66) against GTDB v214.

### *dnaK* and *gyrB* search in metagenome-assembled contigs

To assess the presence of *dnaK* and *gyrB* in the assembled metagenomes, we used all contigs with a length ≥2,000 bp, regardless of whether they had been binned into a MAG or not. We used Prokka 1.12 (31) to perform gene calling and annotated genes with eggNOG mapper v.2.1.6 (32). Gene annotation tables were ﬁltered as follows: for *dnaK*, we retained entries with preferred name: *dnaK*, PFAMs: HSP70 or eggNOG-OG: COG0443. For *gyrB*, we used the preferred name: *gyrB*, PFAMs: gyrase B, or eggNOG-OG: COG0187.

### Phylogenetic and genomic characterization of *Vescimonas* MAGs

We performed a phylogenetic and genomic characterization of 120 MAGs taxonomically classiﬁed as members of the genus *Vescimonas* from the Colombian cohort. As a reference, we retrieved 73 genomes corresponding to the representative accession of each species of the *Vescimonas* genus in the GTDB (Table S6). We used PhyloPhlAn v.3.0.2 (67) to construct a maximum-likelihood phylogenomic tree of all Colombian and reference MAGs using a concatenated alignment of multiple universally distributed single-copy marker genes. As an outgroup, we used genomes from GTDB representatives of the genus *ER4* (family *Oscillospiraceae*). We visualized the phylogeny using iTOL (68). We performed gene calling using Prokka 1.12 (31), and the predicted proteome was annotated using eggNOG mapper v.2.1.6 (32). We extracted the annotation of the predicted CAZymes from the eggNOG mapper results. Microbial traits were predicted using a python3 implementation of Traitar v.3 (29) (available at https://github.com/nick-youngblut/traitar3) and PhenDB (30) (available at https://phendb.org/).

## Data Availability

DNA reads for 16S rRNA, dnaK, and gyrB are available at the NCBI’s Short Read Archive (BioProject PRJNA417579). The assembled MAGs are available at the European Nucleotide Archive (project PRJEB58436). The R code and associated files to reproduce statistical analyses are available at https://github.com/Vidarium/clostridia.

## SUPPLEMENTAL MATERIAL

Figure S1 msystems.00627-24-s0001.tiff Association of pairwise comparisons among Clostridia.

Figure S2 msystems.00627-24-s0002.tiff Taxonomic mapping of the 120 MAGs assembled from Colombian subjects related to GTDB species representatives.

Figure S3 msystems.00627-24-s0003.tiff Associations between alpha diversity and BMI for the *Vescimonas* MAGs.

Figure S4 msystems.00627-24-s0004.tiff Associations between the abundance (rarefied counts) of *Vescimonas* and BMI.

Figure S5 msystems.00627-24-s0005.tiff Distribution prevalence and counts of ASVs.

Supplemental tables msystems.00627-24-s0006.xlsx Tables S1-S8.

ASM does not own the copyrights to Supplemental Material that may be linked to, or accessed through, an article. The authors have granted ASM a non-exclusive, world-wide license to publish the Supplemental Material files. Please contact the corresponding author directly for reuse.

## References

[1] Oren A, Garrity GM. 2021. Valid publication of the names of forty-two phyla of prokaryotes. Int J Syst Evol Microbiol 71:005056. doi:10.1099/ijsem.0.005056 34694987

[2] Vital M, Karch A, Pieper DH. 2017. Colonic butyrate-producing communities in humans: an overview using omics data. mSystems 2:e00130-17. doi:10.1128/mSystems.00130-17 29238752 PMC5715108

[3] Pasolli E, Asnicar F, Manara S, Zolfo M, Karcher N, Armanini F, Beghini F, Manghi P, Tett A, Ghensi P, Collado MC, Rice BL, DuLong C, Morgan XC, Golden CD, Quince C, Huttenhower C, Segata N. 2019. Extensive unexplored human microbiome diversity revealed by over 150,000 genomes from metagenomes spanning age, geography, and lifestyle. Cell 176:649–662. doi:10.1016/j.cell.2019.01.001 30661755 PMC6349461

[4] Piquer-Esteban S, Ruiz-Ruiz S, Arnau V, Diaz W, Moya A. 2022. Exploring the universal healthy human gut microbiota around the world. Comput Struct Biotechnol J 20:421–433. doi:10.1016/j.csbj.2021.12.035 35035791 PMC8749183

[5] Louis P, Flint HJ. 2009. Diversity, metabolism and microbial ecology of butyrate-producing bacteria from the human large intestine. FEMS Microbiol Lett 294:1–8. doi:10.1111/j.1574-6968.2009.01514.x 19222573

[6] Vital M, Howe AC, Tiedje JM. 2014. Revealing the bacterial butyrate synthesis pathways by analyzing (meta) genomic data. mBio 5:e00889. doi:10.1128/mBio.00889-14 24757212 PMC3994512

[7] Lopetuso LR, Scaldaferri F, Petito V, Gasbarrini A. 2013. Commensal Clostridia: leading players in the maintenance of gut homeostasis. Gut Pathog 5:23. doi:10.1186/1757-4749-5-23 23941657 PMC3751348

[8] Almeida A, Nayfach S, Boland M, Strozzi F, Beracochea M, Shi ZJ, Pollard KS, Sakharova E, Parks DH, Hugenholtz P, Segata N, Kyrpides NC, Finn RD. 2021. A unified catalog of 204,938 reference genomes from the human gut microbiome. Nat Biotechnol 39:105–114. doi:10.1038/s41587-020-0603-3 32690973 PMC7801254

[9] de la Cuesta-Zuluaga J, Corrales-Agudelo V, Velásquez-Mejía EP, Carmona JA, Abad JM, Escobar JS. 2018. Gut microbiota is associated with obesity and cardiometabolic disease in a population in the midst of Westernization. Sci Rep 8:11356. doi:10.1038/s41598-018-29687-x 30054529 PMC6063892

[10] Atarashi K, Tanoue T, Oshima K, Suda W, Nagano Y, Nishikawa H, Fukuda S, Saito T, Narushima S, Hase K, Kim S, Fritz JV, Wilmes P, Ueha S, Matsushima K, Ohno H, Olle B, Sakaguchi S, Taniguchi T, Morita H, Hattori M, Honda K. 2013. Treg induction by a rationally selected mixture of Clostridia strains from the human microbiota. Nature 500:232–236. doi:10.1038/nature12331 23842501

[11] Peters BA, Shapiro JA, Church TR, Miller G, Trinh-Shevrin C, Yuen E, Friedlander C, Hayes RB, Ahn J. 2018. A taxonomic signature of obesity in a large study of American adults. Sci Rep 8:9749. doi:10.1038/s41598-018-28126-1 29950689 PMC6021409

[12] Forbes JD, Chen C-Y, Knox NC, Marrie R-A, El-Gabalawy H, de Kievit T, Alfa M, Bernstein CN, Van Domselaar G. 2018. A comparative study of the gut Microbiota in immune-mediated inflammatory diseases—does a common dysbiosis exist? Microbiome 6:221. doi:10.1186/s40168-018-0603-4 30545401 PMC6292067

[13] Michail S, Durbin M, Turner D, Griffiths AM, Mack DR, Hyams J, Leleiko N, Kenche H, Stolfi A, Wine E. 2012. Alterations in the gut microbiome of children with severe ulcerative colitis. Inflamm Bowel Dis 18:1799–1808. doi:10.1002/ibd.22860 22170749 PMC3319508

[14] Geistlinger L, Mirzayi C, Zohra F, Azhar R, Elsafoury S, Grieve C, Wokaty J, Gamboa-Tuz SD, Sengupta P, Hecht I, Ravikrishnan A, Gonçalves RS, Franzosa E, Raman K, Carey V, Dowd JB, Jones HE, Davis S, Segata N, Huttenhower C, Waldron L. 2024. BugSigDB captures patterns of differential abundance across a broad range of host-associated microbial signatures. Nat Biotechnol 42:790–802. doi:10.1038/s41587-023-01872-y 37697152 PMC11098749

[15] Tringe SG, Hugenholtz P. 2008. A renaissance for the pioneering 16S rRNA gene. Curr Opin Microbiol 11:442–446. doi:10.1016/j.mib.2008.09.011 18817891

[16] Caporaso JG, Lauber CL, Walters WA, Berg-Lyons D, Lozupone CA, Turnbaugh PJ, Fierer N, Knight R. 2011. Global patterns of 16S rRNA diversity at a depth of millions of sequences per sample. Proc Natl Acad Sci USA 108 Suppl 1:4516–4522. doi:10.1073/pnas.1000080107 20534432 PMC3063599

[17] Caro-Quintero A, Ochman H. 2015. Assessing the unseen bacterial diversity in microbial communities. Genome Biol Evol 7:3416–3425. doi:10.1093/gbe/evv234 26615218 PMC4700968

[18] Moeller AH, Caro-Quintero A, Mjungu D, Georgiev AV, Lonsdorf EV, Muller MN, Pusey AE, Peeters M, Hahn BH, Ochman H. 2016. Cospeciation of gut microbiota with hominids. Science 353:380–382. doi:10.1126/science.aaf3951 27463672 PMC4995445

[19] La Reau AJ, Meier-Kolthoff JP, Suen G. 2016. Sequence-based analysis of the genus Ruminococcus resolves its phylogeny and reveals strong host association. Microb Genom 2:e000099. doi:10.1099/mgen.0.000099 28348838 PMC5359413

[20] Guo J, Cole J, Brown CT, Tiedje J. 2018. Comparing faster evolving rplB and rpsC versus SSU rRNA for improved microbial community resolution:bioRxiv. doi:10.1101/435099

[21] De Filippis F, Pasolli E, Tett A, Tarallo S, Naccarati A, De Angelis M, Neviani E, Cocolin L, Gobbetti M, Segata N, Ercolini D. 2019. Distinct genetic and functional traits of human intestinal Prevotella copri strains are associated with different habitual diets. Cell Host Microbe 25:444–453. doi:10.1016/j.chom.2019.01.004 30799264

[22] Olm MR, Crits-Christoph A, Diamond S, Lavy A, Matheus Carnevali PB, Banfield JF. 2020. Consistent metagenome-derived metrics verify and delineate bacterial species boundaries. mSystems 5:e00731-19. doi:10.1128/mSystems.00731-19 31937678 PMC6967389

[23] de la Cuesta-Zuluaga J, Corrales-Agudelo V, Carmona JA, Abad JM, Escobar JS. 2018. Body size phenotypes comprehensively assess cardiometabolic risk and refine the association between obesity and gut microbiota. Int J Obes 42:424–432. doi:10.1038/ijo.2017.281 29142244

[24] de la Cuesta-Zuluaga J, Ley RE, Youngblut ND. 2020. Struo: a pipeline for building custom databases for common metagenome profilers. Bioinformatics 36:2314–2315. doi:10.1093/bioinformatics/btz899 31778148

[25] Youngblut ND, de la Cuesta-Zuluaga J, Ley RE. 2022. Incorporating genome-based phylogeny and functional similarity into diversity assessments helps to resolve a global collection of human gut metagenomes. Environ Microbiol 24:3966–3984. doi:10.1111/1462-2920.15910 35049120

[26] Love MI, Huber W, Anders S. 2014. Moderated estimation of fold change and dispersion for RNA-seq data with DESeq2. Genome Biol 15:550. doi:10.1186/s13059-014-0550-8 25516281 PMC4302049

[27] Kitahara M, Shigeno Y, Shime M, Matsumoto Y, Nakamura S, Motooka D, Fukuoka S, Nishikawa H, Benno Y. 2021. Vescimonas gen. nov., Vescimonas coprocola sp. nov., Vescimonas fastidiosa sp. nov., Pusillimonas gen. nov. and Pusillimonas faecalis sp. nov. isolated from human faeces. Int J Syst Evol Microbiol 71. doi:10.1099/ijsem.0.005066 34726590

[28] de la Cuesta-Zuluaga J, Huus KE, Youngblut ND, Escobar JS, Ley RE. 2023. Obesity is the main driver of altered gut microbiome functions in the metabolically unhealthy. Gut Microbes 15:2246634. doi:10.1080/19490976.2023.2246634 37680093 PMC10486298

[29] Aaron W, Kyra M, Jeremy F, Phillip BP, Andreas B, MA C. 2016. From genomes to phenotypes: traitar, the microbial trait analyzer. mSystems 1:e00101-16. doi:10.1128/mSystems.00101-16 28066816 PMC5192078

[30] Feldbauer R, Schulz F, Horn M, Rattei T. 2015. Prediction of microbial phenotypes based on comparative genomics. BMC Bioinformatics 16 Suppl 14:S1. doi:10.1186/1471-2105-16-S14-S1 PMC4603748 26451672

[31] Seemann T. 2014. Prokka: rapid prokaryotic genome annotation. Bioinformatics 30:2068–2069. doi:10.1093/bioinformatics/btu153 24642063

[32] Cantalapiedra CP, Hernández-Plaza A, Letunic I, Bork P, Huerta-Cepas J. 2021. eggNOG-mapper v2: functional annotation, orthology assignments, and domain prediction at the metagenomic scale. Mol Biol Evol 38:5825–5829. doi:10.1093/molbev/msab293 34597405 PMC8662613

[33] De Filippis F, Pasolli E, Ercolini D. 2020. Newly explored Faecalibacterium diversity is connected to age, lifestyle, geography, and disease. Curr Biol 30:4932–4943. doi:10.1016/j.cub.2020.09.063 33065016

[34] Lloyd-Price J, Mahurkar A, Rahnavard G, Crabtree J, Orvis J, Hall AB, Brady A, Creasy HH, McCracken C, Giglio MG, McDonald D, Franzosa EA, Knight R, White O, Huttenhower C. 2017. Strains, functions and dynamics in the expanded human microbiome project. Nature 550:61–66. doi:10.1038/nature23889 28953883 PMC5831082

[35] Youngblut ND, Reischer GH, Dauser S, Maisch S, Walzer C, Stalder G, Farnleitner AH, Ley RE. 2021. Vertebrate host phylogeny influences gut archaeal diversity. Nat Microbiol 6:1443–1454. doi:10.1038/s41564-021-00980-2 34702978 PMC8556154

[36] Li J, Zhao F, Wang Y, Chen J, Tao J, Tian G, Wu S, Liu W, Cui Q, Geng B, Zhang W, Weldon R, Auguste K, Yang L, Liu X, Chen L, Yang X, Zhu B, Cai J. 2017. Gut microbiota dysbiosis contributes to the development of hypertension. Microbiome 5:14. doi:10.1186/s40168-016-0222-x 28143587 PMC5286796

[37] Gacesa R, Kurilshikov A, Vich Vila A, Sinha T, Klaassen MAY, Bolte LA, Andreu-Sánchez S, Chen L, Collij V, Hu S, et al.. 2020. The Dutch microbiome project defines factors that shape the healthy gut microbiome. doi:10.1101/2020.11.27.401125

[38] Yan Y, Nguyen LH, Franzosa EA, Huttenhower C. 2020. Strain-level epidemiology of microbial communities and the human microbiome. Genome Med 12:71. doi:10.1186/s13073-020-00765-y 32791981 PMC7427293

[39] Konikoff T, Gophna U. 2016. Oscillospira: a central, enigmatic component of the human gut microbiota. Trends Microbiol 24:523–524. doi:10.1016/j.tim.2016.02.015 26996766

[40] Yang J, Li Y, Wen Z, Liu W, Meng L, Huang H. 2021. Oscillospira - a candidate for the next-generation probiotics. Gut Microbes 13:1987783. doi:10.1080/19490976.2021.1987783 34693878 PMC8547878

[41] Leylabadlo HE, Ghotaslou R, Feizabadi MM, Farajnia S, Moaddab SY, Ganbarov K, Khodadadi E, Tanomand A, Sheykhsaran E, Yousefi B, Kafil HS. 2020. The critical role of Faecalibacterium prausnitzii in human health: an overview. Microb Pathog 149:104344. doi:10.1016/j.micpath.2020.104344 32534182

[42] Hiseni P, Rudi K, Wilson RC, Hegge FT, Snipen L. 2021. HumGut: a comprehensive human gut prokaryotic genomes collection filtered by metagenome data. Microbiome 9:165. doi:10.1186/s40168-021-01114-w 34330336 PMC8325300

[43] Companys J, Gosalbes MJ, Pla-Pagà L, Calderón-Pérez L, Llauradó E, Pedret A, Valls RM, Jiménez-Hernández N, Sandoval-Ramirez BA, Del Bas JM, Caimari A, Rubió L, Solà R. 2021. Gut microbiota profile and its association with clinical variables and dietary intake in overweight/obese and lean subjects: a cross-sectional study. Nutrients 13:2032. doi:10.3390/nu13062032 34199239 PMC8231825

[44] Mirzayi C, Renson A, Zohra F, Elsafoury S, Geistlinger L, Kasselman LJ, Eckenrode K, van de Wijgert J, Loughman A, Marques FZ, et al.. 2021. Reporting guidelines for human microbiome research: the STORMS checklist. Nat Med 27:1885–1892. doi:10.1038/s41591-021-01552-x 34789871 PMC9105086

[45] Thompson JD, Higgins DG, Gibson TJ. 1994. CLUSTAL W: improving the sensitivity of progressive multiple sequence alignment through sequence weighting, position-specific gap penalties and weight matrix choice. Nucleic Acids Res 22:4673–4680. doi:10.1093/nar/22.22.4673 7984417 PMC308517

[46] Suyama M, Torrents D, Bork P. 2006. PAL2NAL: robust conversion of protein sequence alignments into the corresponding codon alignments. Nucleic Acids Res 34:W609–12. doi:10.1093/nar/gkl315 16845082 PMC1538804

[47] Bolger AM, Lohse M, Usadel B. 2014. Trimmomatic: a flexible trimmer for illumina sequence data. Bioinformatics 30:2114–2120. doi:10.1093/bioinformatics/btu170 24695404 PMC4103590

[48] Magoč T, Salzberg SL. 2011. FLASH: fast length adjustment of short reads to improve genome assemblies. Bioinformatics 27:2957–2963. doi:10.1093/bioinformatics/btr507 21903629 PMC3198573

[49] Shen W, Le S, Li Y, Hu F. 2016. SeqKit: a cross-platform and ultrafast toolkit for FASTA/Q file manipulation. PLoS One 11:e0163962. doi:10.1371/journal.pone.0163962 27706213 PMC5051824

[50] Callahan BJ, McMurdie PJ, Rosen MJ, Han AW, Johnson AJA, Holmes SP. 2016. DADA2: high-resolution sample inference from illumina amplicon data. Nat Methods 13:581–583. doi:10.1038/nmeth.3869 27214047 PMC4927377

[51] Bolyen E, Rideout JR, Dillon MR, Bokulich NA, Abnet CC, Al-Ghalith GA, Alexander H, Alm EJ, Arumugam M, Asnicar F, et al.. 2019. Reproducible, interactive, scalable and extensible microbiome data science using QIIME 2. Nat Biotechnol 37:852–857. doi:10.1038/s41587-019-0209-9 31341288 PMC7015180

[52] Callahan BJ, McMurdie PJ, Holmes SP. 2017. Exact sequence variants should replace operational taxonomic units in marker-gene data analysis. ISME J 11:2639–2643. doi:10.1038/ismej.2017.119 28731476 PMC5702726

[53] Katoh K, Standley DM. 2013. MAFFT multiple sequence alignment software version 7: improvements in performance and usability. Mol Biol Evol 30:772–780. doi:10.1093/molbev/mst010 23329690 PMC3603318

[54] Paradis E, Schliep K. 2019. Ape 5.0: an environment for modern phylogenetics and evolutionary analyses in R. Bioinformatics 35:526–528. doi:10.1093/bioinformatics/bty633 30016406

[55] R Core Team. 2019. R: a language and environment for statistical computing. R Foundation for Statistical Computing, Vienna, Austria.

[56] McMurdie PJ, Holmes S. 2013. Phyloseq: an R package for reproducible interactive analysis and graphics of microbiome census data. PLoS One 8:e61217. doi:10.1371/journal.pone.0061217 23630581 PMC3632530

[57] Karasov TL, Almario J, Friedemann C, Ding W, Giolai M, Heavens D, Kersten S, Lundberg DS, Neumann M, Regalado J, Neher RA, Kemen E, Weigel D. 2018. Arabidopsis thaliana and Pseudomonas pathogens exhibit stable associations over evolutionary timescales. Cell Host Microbe 24:168–179. doi:10.1016/j.chom.2018.06.011 30001519 PMC6054916

[58] Droop AP. 2016. fqtools: an efficient software suite for modern FASTQ file manipulation. Bioinformatics 32:1883–1884. doi:10.1093/bioinformatics/btw088 27153699 PMC4908325

[59] Jiang H, Lei R, Ding S-W, Zhu S. 2014. Skewer: a fast and accurate adapter trimmer for next-generation sequencing paired-end reads. BMC Bioinformatics 15:182. doi:10.1186/1471-2105-15-182 24925680 PMC4074385

[60] Rodriguez-R LM, Gunturu S, Tiedje JM, Cole JR, Konstantinidis KT. 2018. Nonpareil 3: fast estimation of metagenomic coverage and sequence diversity. mSystems 3:e00039-18. doi:10.1128/mSystems.00039-18 29657970 PMC5893860

[61] Youngblut ND, de la Cuesta-Zuluaga J, Reischer GH, Dauser S, Schuster N, Walzer C, Stalder G, Farnleitner AH, Ley RE. 2020. Large-scale metagenome assembly reveals novel animal-associated microbial genomes, biosynthetic gene clusters, and other genetic diversity. mSystems 5:e01045-20. doi:10.1128/mSystems.01045-20 33144315 PMC7646530

[62] Cepeda V, Liu B, Almeida M, Hill CM, Koren S, Treangen TJ, Pop M. 2017. MetaCompass: reference-guided assembly of metagenomes. Bioinformatics. doi:10.1101/212506 PMC12570322 41015028

[63] Nurk S, Meleshko D, Korobeynikov A, Pevzner PA. 2017. metaSPAdes: a new versatile metagenomic assembler. Genome Res 27:824–834. doi:10.1101/gr.213959.116 28298430 PMC5411777

[64] Sieber CMK, Probst AJ, Sharrar A, Thomas BC, Hess M, Tringe SG, Banfield JF. 2018. Recovery of genomes from metagenomes via a dereplication, aggregation and scoring strategy. Nat Microbiol 3:836–843. doi:10.1038/s41564-018-0171-1 29807988 PMC6786971

[65] Olm MR, Brown CT, Brooks B, Banfield JF. 2017. dRep: a tool for fast and accurate genomic comparisons that enables improved genome recovery from metagenomes through de-replication. ISME J 11:2864–2868. doi:10.1038/ismej.2017.126 28742071 PMC5702732

[66] Chaumeil P-A, Mussig AJ, Hugenholtz P, Parks DH. 2019. GTDB-Tk: a toolkit to classify genomes with the genome taxonomy database. Bioinformatics 36:1925–1927. doi:10.1093/bioinformatics/btz848 31730192 PMC7703759

[67] Asnicar F, Thomas AM, Beghini F, Mengoni C, Manara S, Manghi P, Zhu Q, Bolzan M, Cumbo F, May U, Sanders JG, Zolfo M, Kopylova E, Pasolli E, Knight R, Mirarab S, Huttenhower C, Segata N. 2020. Precise phylogenetic analysis of microbial isolates and genomes from metagenomes using phyloPhlAn 3.0. Nat Commun 11:2500. doi:10.1038/s41467-020-16366-7 32427907 PMC7237447

[68] Letunic I, Bork P. 2016. Interactive tree of life (iTOL) V3: an online tool for the display and annotation of phylogenetic and other trees. Nucleic Acids Res 44:W242–5. doi:10.1093/nar/gkw290 27095192 PMC4987883

