# Supplementary figures and images for: Gut microbiome diversity within Clostridia is negatively associated with human obesity
Source: mSystems. 2024 Jul 16;9(8):e00627-24. doi: 10.1128/msystems.00627-24 (PMC11334427; doi:10.1128/msystems.00627-24)

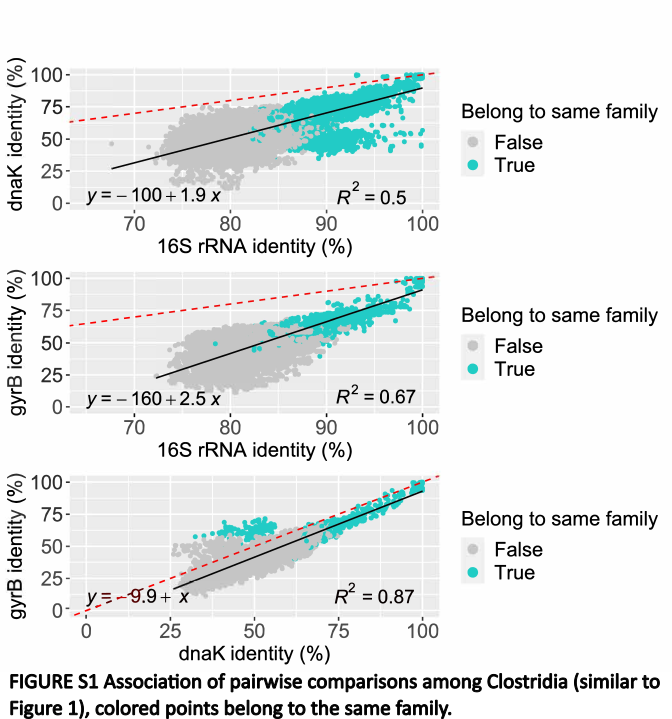

Supplement: Figure S1 — Association of pairwise comparisons among Clostridia. [file msystems.00627-24-s0001.tiff]

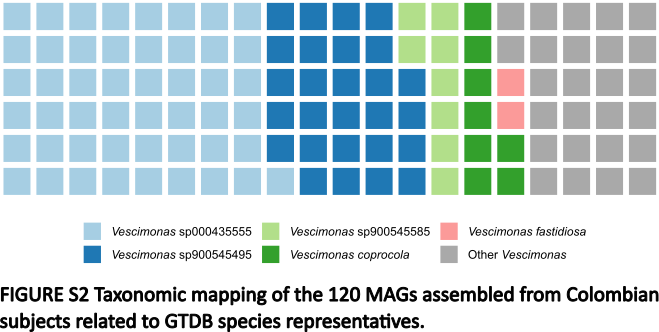

Supplement: Figure S2 — Taxonomic mapping of the 120 MAGs assembled from Colombian subjects related to GTDB species representatives. [file msystems.00627-24-s0002.tiff]

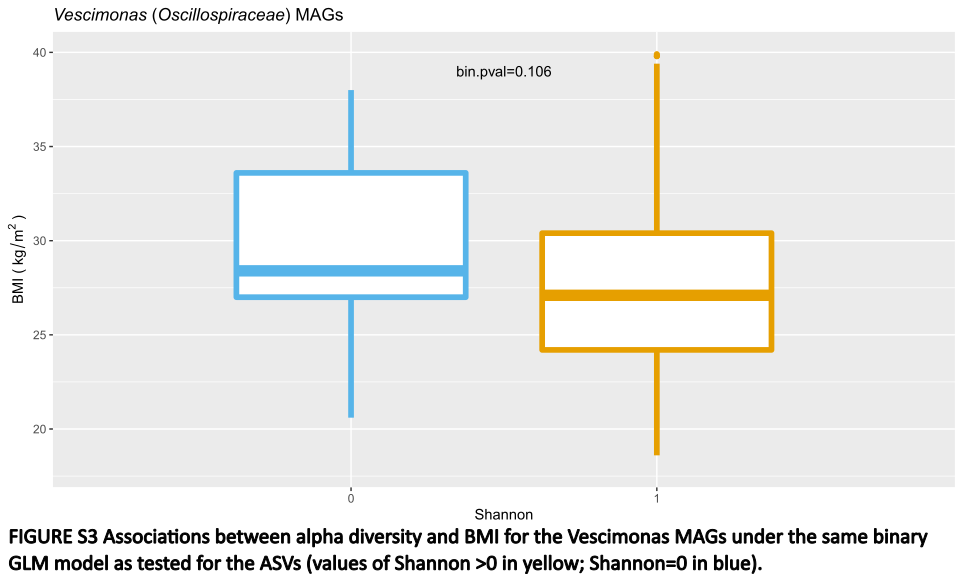

Supplement: Figure S3 — Associations between alpha diversity and BMI for the Vescimonas MAGs. [file msystems.00627-24-s0003.tiff]

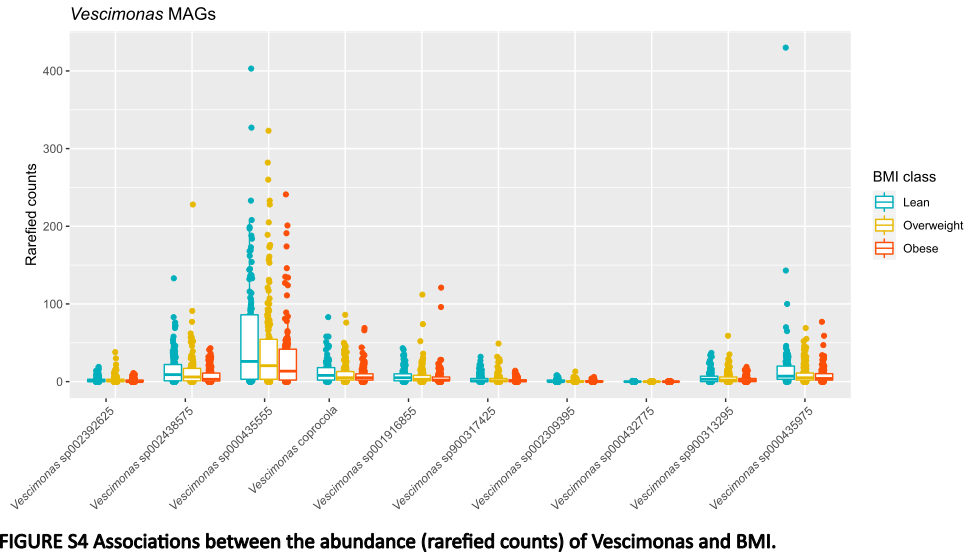

Supplement: Figure S4 — Associations between the abundance (rarefied counts) of Vescimonas and BMI. [file msystems.00627-24-s0004.tiff]

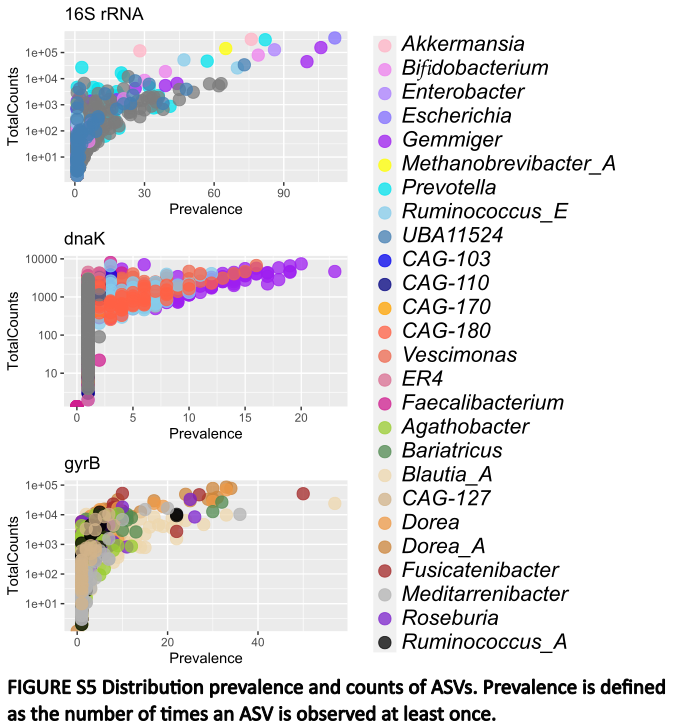

Supplement: Figure S5 — Distribution prevalence and counts of ASVs. [file msystems.00627-24-s0005.tiff]
